# Characteristics of COPD phenotypes classified according to the findings of HRCT and spirometric indices and its correlation to clinical characteristics

**DOI:** 10.4314/ahs.v18i1.13

**Published:** 2018-03

**Authors:** Ravi Bhaskar, Seema Singh, Pooja Singh

**Affiliations:** Department of Pulmonary Medicine, Career Institute of Medical Sciences, Lucknow, (UP) India

**Keywords:** Chronic obstructive pulmonary disease, high resolution computed tomography, spirometry

## Abstract

**Introduction:**

In recent years, there has been increasing interest in diagnosing various components of chronic obstructive pulmonary disease (COPD) using high-resolution computed tomography (HRCT). The present study was undertaken to evaluate HRCT features in patients with COPD.

**Materials and methods:**

Fifty patients of COPD (confirmed on Spirometry as per the GOLD guidelines 2014 guidelines) were enrolled, out of which 35 patients got a HRCT done. The Philips computer program for lung densitometry was used with these limits (−800/−1, 024 Hounsfield unit [HU]) to calculate densities, after validating densitometry values with phantoms. We established the area with a free hand drawing of the region of interest, then we established limits (in HUs) and the computer program calculated the attenuation as mean lung density (MLD) of the lower and upper lobes.

**Results:**

There was a significant correlation between smoking index and anteroposterior tracheal diameter (P = 0.036). Tracheal index was found to be decreasing with increasing disease severity which was statistically significant (P = 0.037). A mild linear correlation of pre-forced expiratory volume in the first second (FEV1) was observed with lower lobe and total average MLD while a mild linear correlation of post-FEV1 was observed with both coronal (P = 0.042) and sagittal (P = 0.001) lower lobes MLD. In addition, there was a linear correlation between both pre (P = 0.050) and post (P = 0.024) FEV1/forced vital capacity with sagittal lower lobe MLD.

**Conclusion:**

HRCT may be an important additional tool in the holistic evaluation of COPD.

## Introduction

COPD is a chronic inflammatory disease affecting the airways, leading to significant morbidity and mortality throughout the world. The prevalence of stage II or higher COPD is 10.1% worldwide^1^. COPD is a heterogeneous condition with multiple clinical and functional profiles. It consists of a number of different pathological processes, which are modified by varied host susceptibility. Forced expiratory volume in 1 second (FEV1) is not enough to describe this heterogeneity, especially in severe COPD patients. But, there is as yet no clear alternative to characterize the many faces of COPD. High-resolution computed tomography (HRCT) of the chest helps determine which structures are more involved (airways or lung parenchyma) and to quantify the damage. Besides, some authors have been using the method to propose a tomography phenotyping of COPD. Quantitative assessment of EMP by CT scanning has a good correlation with airflow obstruction.^2^–^4^ Objective measures of airway wall thickening also correlated well with lung function.^4^–^6^ whether the presence of this specific lung structural abnormalities (EMP, airway wall thickening, and/or bronchiectasis) predict meaningful clinical outcome is not known.^7^ Some authors compared CT visual assessment of COPD patients with quantitative analysis and several physiological parameters and found a good correlation.^8^ Besides, Kitaguchi et al^9^ used CT visual assessment to phenotype COPD patients, and this method allows the recognition of subsets of patients with clinical and functional distinct characteristics.

For establishing a diagnosis of COPD, according to the Global Initiative for Chronic Obstructive Lung Diseases^1^ guidelines, spirometry showing fixed airflow obstruction is essential. Spirometry[a1] is an inexpensive, easily reproducible and readily available test. But there are certain drawbacks of spirometry, for instance, it cannot be used in patients presenting for the first time in an exacerbation (being difficult to perform and less reliable). Also, spirometry is an effort dependent procedure, so elderly patients and patients with neurological or psychiatric disorders who are unable to follow commands face difficulty in performing spirometry. Patients with oro-facial trauma or tumors are not able to perform too. So, in these groups of patients, an alternative test to spirometry is required for establishing a diagnosis of COPD.

There is the need to have a holistic evaluation of COPD patients, other than just measuring the level of obstruction as done by spirometry. HRCT scan of thorax fulfills this requirement. It provides information about the extent and distribution of emphysema, the presence of chronic bronchitis or other associated findings such as bullae, bronchiectasis and cysts. Also, it is essential in the patients of COPD for ruling out alternative diagnoses and for pre-surgical assessment before lung volume reduction surgeries or bullectomy. There is increasing role of HRCT in evaluation of early emphysema in asymptomatic smokers, in patients of chronic bronchitis and in assessment of the various phenotypes of COPD.

The purpose of this study was to investigate the usefulness of High Resolution Computed Tomography (HRCT) in the patients with Chronic Obstructive Pulmonary Disease (COPD) and relate it to clinical and spirometric values for future correlation and derive a means for evaluation of obstruction in absence of spirometry.

## Materials and methods

It was a prospective observational study carried out at a tertiary care teaching hospital of North India. Patients were recruited from outdoor patients department of Pulmonary Medicine, Career Institute of Medical Sciences, Lucknow. They had undergone a detailed questionnaire and after that spirometry was performed of every individual and the patients were enrolled finally according to the GOLD criteria (in accordance with current GOLD guidlenes)^1^ COPD was defined by a post-bronchodilator-FEV1/FVC ratio < 0.70. COPD severity was determined by the GOLD criteria. Subjects with a post-bronchodilator FEV1/FVC ratio ≥ 0.70 were considered not to have COPD) in the study. The duration of study March 2016 to April 2017. Total 50 newly diagnosed patients suffering from COPD attending the department of pulmonary medicine as per GOLD guidelines 2016 were included in the study.^1^ The written informed consent was obtained fulfilling the inclusion and exclusion criteria of the study. The study was approved by the institutional ethics committee. Out of a total of 50 patients, 35 patients got a HRCT done, and were included in the study after an explicit, written consent. Patient's detailed history was taken, including smoking status and pack years. They[a2] underwent physical examination, routine blood investigations including arterial blood gas (ABG) analysis and a chest radiograph (postero-anterior view). The spirometry was carried out on Morgan Medical Private Limited-PULMOLAB 435 (SPIRO 232). Spirometric indices were measured using the best out of 3 satisfactory performances The[a3] parameters recorded were FEV1 (forced expiratory volume in the first second) in liters, FVC (forced vital capacity) in liters and FEV1/FVC% (forced expiratory volume in first second/forced vital capacity).

HRCT was carried out using GE CT/e Single Slice Spiral Computed Tomography machine, without contrast. The features assessed on HRCT were tracheal index (TI) (ratio of transverse to AP diameter of trachea 1 cm above the aortic arch), thoracic cage ratio (TCR)( ratio of AP to transverse diameter at carina (TCRC) and 5 cm below carina (TCR5C), sterno-aortic distance (SAD) (distance from posterior surface of sternum to anterior margin of aorta at the level of carina, vascular attenuation (thinning and decreased number of pulmonary vessels) and other features such as bronchiectasis, cysts, bullae, pulmonary hypertension, evidence of fibrosis and mediastinal lymphadenopathy. The Philips computer program for lung densitometry was used with these limits (−800/−1,024HU) to calculate densities, after validating densitometry values with phantoms. We established the area with a freehand drawing of the region of interest, then we established limits (in HU) and the computer program calculated the attenuation as mean lung density (MLD) of the lower and upper lobes.

Patients[a4] requiring mechanical ventilation or those having co-existing cardiac disorder leading to breathlessness such as congestive heart failure, cardiomyopathy or coronary artery disease were excluded from the study. The written informed consent was obtained after fulfilling the inclusion and exclusion criteria of the study. The study was approved by the institutional ethics committee.

### Statistical analyses

In an attempt to calculate the severity of disease (FEV1) on the basis of MLD, a predictive model was prepared where FEV1% was considered as a dependent variable while MLD of coronal right upper lobe (CRUL), right lower lobe (CRLL), left upper lobe (CLUL), left lower lobe (CLLL) and sagittal right upper lobe (SRUL), right lower lobe (SRLL), left upper lobe (SLUL) and left lower lobe (SLLL) were taken as an independent variable. The MLD of sagittal RUL, sagittal RLL and sagittal LLL were found to be significantly associated with the dependent variable (p<0.05) and the model had a fair explanatory ability (r2=0.439). Using this model, FEV1 can be calculated as: FEV1% = −44.700 + 0.184 CRUL − 0.230 CRLL − 0.106 CLUL + 0.219 CLLL − 0.350 SRUL + 0.439 SRLL + 0.173 SLUL − 0.218 SLLL.[SK5] The 95% (CI) confidence interval were taken in whole study.

## Results

35 34 (97.14%) had bronchial breathing on auscultation. A sign of heart failure, namely pedal edema was noticed in 7 (20.0%) patients, raised jugular venous pressure (JVP) in 8 (22.8%) patients, tender hepatomegaly in 3 (8.57%) patients and basal crepitations in 9 (25.71%) patients. These features were found more frequently in stage III and IV than stage I and II.

While comparing hematological and biochemical parameters, 4 (11.4%) patients had polycythemia, while 15 patients (15 {42.88%}) had anemia. Among other parameters, values of serum creatinine were found to be significantly increasing with disease severity. On evaluating oxygenation status, more than 50% patients were found to be hypoxic on ABG analysis (54.28%), while 12 (34.28%) patients had hypercapnia. No other correlation was found between blood gases and disease severity. On chest radiography, signs of hyperinflation which were subjectively assessed were flattening of diaphragm in 22 patients (62.58%), a tubular heart in 20 (57.14%) and pruning of vascular markings in 18 (51.4%) patients. 4 (11.43%) patients had bullae identified on chest radiograph and 5 (14.28%) patients had prominent pulmonary trunks.

Among HRCT features (Table 1), vascular attenuation and emphysema were the most common findings (n=31, 88.57%, each). Our study revealed bronchiectasis in a significant proportion of patients (n=19 {54.28%}). Also, patients with bronchiectasis were more often smokers and had lower FEV1. Regarding signs of hyperinflation on HRCT, our study found a mean tracheal index of 0.96(range=0.36–1.95). Saber sheath trachea was seen in 2(5.71%) patients. Mean TCR at carina was 0.77, while mean TCR at a level 5 cm below the carina was 0.69. Increased thoracic cage ratio was found in 11(31.4%) patients, while barrel shaped chest (TCR>0.9) was seen in 6(17.1%) patients.

**Table 1:** Demographic profile and clinical charactereristics of Chronic Obstructive Pulmonary Disease

CT parameter (TOTAL N=35)	No. of patients	% of subjects
Saber Sheath Trachea	2	5.71
Tcr At Carina> 0.75	11	31.42
TCR 5 Cm BELOW CARINA> 0.75	12	25.80
SAD>4 Cm	2	5.71
Vascular Attenuation	31	88.57
Mosaicattenuation Pattern	8	22.85
Bullae	10	28.57
Emphysema	31	88.57
Bronchiectasis	19	54.28
Cyst	6	17.14
Pulmonary Hypertension	4	11.42
Fibrosis	22	62.85
Barrel Shaped Chest	6	17.14

Mean SAD was 2.87 cm (range=1.13–4.26). SAD of >4 cm was seen in 2 (5.71%) patients. In our study, there was significant correlation between smoking index and AP tracheal diameter (p=0.036) (Table 2A & 2B). Tracheal index was found to be decreasing with increasing disease severity (GOLD stage) and this was statistically significant (p=0.037). In our study, mean upper lobe MLD was −839.27 HU, mean lower lobe MLD was −834.91 HU and the mean MLD was −837.08 HU. The lower lobes mean lung density was found to be decreasing with increasing disease severity.

**Table 2A:** Correlation of Thoracic CT Parameters with Patient Characteristics and Pulmonary Function Parameters (Pearson bivariate)

	Tracheal index						Thoracic cage ratio at carina						Thoracic cage ratio 5 cm below carina					
	AP		Trans		Index		AP		Trans		Index		AP		Trans		Index	
	r	p	r	p	r	p	r	p	r	p	r	p	r	p	r	p	r	p
**Age**	−0.06	0.745	0.40	0.017	0.25	0.146	−0.04	0.839	0.25	0.152	−0.20	0.257	0.13	0.461	0.05	0.770	0.10	0.576
**Duration of** **disease**	0.04	0.840	0.05	0.786	−0.03	0.875	−0.07	0.690	0.01	0.937	−0.06	0.738	−0.01	0.939	0.10	0.555	−0.04	0.808
**Smoking index**	0.36	0.036	0.07	0.691	−0.27	0.122	−0.04	0.836	0.17	0.316	−0.11	0.524	0.03	0.878	0.31	0.070	−0.07	0.698
**Pack years**	0.33	0.052	0.05	0.784	−0.26	0.130	−0.05	0.758	0.18	0.305	−0.12	0.485	0.01	0.936	0.30	0.080	−0.08	0.659
MMRC*	−0.10	0.561	0.28	0.100	0.23	0.189	0.00	0.996	0.12	0.509	−0.04	0.832	0.01	0.936	0.17	0.315	−0.05	0.763
GOLD Stage*	−0.15	0.396	0.13	0.455	0.20	0.256	−0.30	0.081	0.09	0.588	−0.16	0.364	−0.26	0.130	0.24	0.171	−0.35	0.042
**Hb**	−0.42	0.013	0.08	0.644	0.43	0.010	−0.04	0.820	−0.08	0.629	0.06	0.716	−0.11	0.513	0.13	0.448	−0.17	0.334
**TLC**	0.26	0.136	0.09	0.605	−0.14	0.418	−0.05	0.794	0.25	0.154	−0.17	0.340	−0.01	0.975	0.07	0.689	−0.04	0.832
**RBS**	−0.10	0.580	0.09	0.595	0.12	0.475	0.07	0.697	−0.33	0.052	0.33	0.055	−0.31	0.074	0.02	0.892	−0.29	0.093
**Urea**	0.11	0.518	0.18	0.312	−0.04	0.809	0.06	0.748	0.07	0.676	0.01	0.962	−0.01	0.932	0.15	0.397	−0.06	0.744
**Creatinine**	−0.07	0.684	0.05	0.787	0.05	0.761	−0.19	0.274	0.09	0.612	−0.09	0.588	−0.24	0.172	0.24	0.156	−0.30	0.080
**Ph**	−0.13	0.449	0.21	0.223	0.22	0.210	−0.01	0.947	−0.01	0.942	0.10	0.555	−0.15	0.375	0.21	0.223	−0.21	0.227
**pO_2_**	−0.05	0.763	−0.04	0.806	−0.06	0.736	0.38	0.025	0.12	0.491	0.10	0.565	0.45	0.006	0.20	0.238	0.35	0.040
**pCO_2_**	−0.18	0.299	0.11	0.544	0.22	0.207	−0.22	0.204	0.07	0.708	−0.18	0.305	−0.11	0.540	−0.07	0.683	−0.08	0.663
**HCO_3_**	−0.23	0.175	0.13	0.449	0.27	0.117	−0.21	0.226	0.07	0.689	−0.13	0.451	−0.16	0.357	0.00	0.982	−0.14	0.409
**SpO_2_**	0.02	0.889	−0.02	0.928	−0.08	0.630	0.40	0.017	−0.07	0.690	0.23	0.184	0.35	0.042	0.03	0.845	0.31	0.066
**FEV1 Pre**	0.09	0.608	−0.31	0.070	−0.29	0.089	0.40	0.017	−0.21	0.227	0.32	0.061	0.24	0.172	−0.17	0.336	0.30	0.085
**FEV1 Post**	0.01	0.966	−0.38	0.024	−0.27	0.113	0.42	0.012	−0.26	0.134	0.36	0.036	0.21	0.231	−0.23	0.179	0.29	0.097
**FVC Pre**	0.03	0.853	−0.16	0.372	−0.12	0.489	0.34	0.047	−0.19	0.285	0.30	0.077	0.14	0.419	−0.07	0.701	0.17	0.342
**FVC Post**	0.03	0.853	−0.07	0.685	−0.06	0.724	0.32	0.059	−0.22	0.198	0.33	0.056	0.10	0.578	−0.10	0.560	0.13	0.452
**V/C Pre**	0.10	0.566	−0.43	0.011	−0.35	0.038	0.24	0.171	−0.20	0.250	0.26	0.135	0.04	0.841	0.00	0.983	0.05	0.793
**V/C Post**	−0.10	0.563	−0.47	0.004	−0.23	0.176	0.26	0.134	−0.19	0.284	0.25	0.145	0.08	0.656	0.00	0.980	0.09	0.619

r<0.3: Negligible correlation; r=0.3–0.5: Mild correlation; r=0.50.7: Moderate correlation; r>0.7: Strong correlation;

* Spearman rank correlation

**Table 2B:** Correlation of Other CT Parameters with Patient Characteristics and Pulmonary Function Parameters (Pearson bivariate)

	SAD		APD		TCSA		Coronal		Coronal		Coronal		Coronal		Sagittal		Sagittal		Sagittal		Sagittal	
							RUL	MLD	RLL	MLD	LUL	MLD	LLL	MLD	RUL	MLD	RLL	MLD	LUL	MLD	LLL	MLD
	r	p	r	p	r	p	r	p	r	p	r	p	r	p	r	p	r	p	r	p	r	p
**Age**	−0.25	0.178	−0.06	0.739	0.10	0.587	−0.02	0.911	−0.12	0.530	−0.10	0.609	−0.10	0.596	0.07	0.723	−0.12	0.526	−0.04	0.832	−0.08	0.650
**Duration of** **disease**	0.23	0.221	−0.03	0.885	0.10	0.605	−0.06	0.740	−0.04	0.833	0.03	0.893	0.12	0.519	0.12	0.523	−0.06	0.735	0.16	0.385	−0.11	0.571
**Smoking** **index**	0.20	0.273	0.08	0.660	0.20	0.290	0.08	0.684	0.06	0.736	0.07	0.703	0.07	0.714	0.10	0.577	0.11	0.540	0.09	0.630	0.05	0.800
**Pack years**	0.22	0.239	0.07	0.725	0.18	0.320	0.11	0.545	0.08	0.658	0.11	0.572	0.10	0.601	0.14	0.462	0.14	0.468	0.13	0.491	0.08	0.660
MMRC*	0.08	0.676	0.02	0.902	0.07	0.702	0.20	0.290	0.11	0.543	0.21	0.257	0.09	0.614	0.22	0.236	0.15	0.412	0.08	0.663	0.07	0.694
**GOLD** Stage*	−0.19	0.307	−0.17	0.351	−0.13	0.495	−0.02	0.912	0.01	0.975	−0.10	0.579	−0.17	0.364	0.11	0.568	0.03	0.874	−0.02	0.899	−0.13	0.485
**Hb**	−0.01	0.961	0.01	0.937	−0.07	0.720	0.11	0.548	0.23	0.220	0.03	0.881	−0.01	0.973	0.31	0.091	0.38	0.036	0.07	0.695	0.05	0.805
**TLC**	0.04	0.846	−0.08	0.650	0.06	0.749	0.16	0.382	0.12	0.524	0.16	0.379	0.00	0.983	0.06	0.731	−0.03	0.865	0.03	0.891	−0.04	0.824
**RBS**	−0.18	0.339	0.09	0.614	−0.24	0.188	−0.35	0.054	−0.34	0.063	−0.50	0.004	−0.40	0.024	−0.27	0.149	−0.19	0.311	−0.29	0.112	−0.28	0.126
**Urea**	0.08	0.668	0.05	0.782	−0.01	0.967	−0.06	0.739	−0.10	0.607	−0.24	0.199	−0.19	0.314	−0.07	0.698	−0.03	0.872	−0.18	0.345	0.02	0.909
**Creatinine**	−0.11	0.538	−0.14	0.468	−0.12	0.536	−0.24	0.201	−0.18	0.324	−0.34	0.062	−0.34	0.061	−0.12	0.508	−0.11	0.572	−0.33	0.072	−0.23	0.222
**Ph**	−0.08	0.666	0.04	0.816	−0.06	0.745	0.18	0.325	0.07	0.722	−0.12	0.523	−0.30	0.105	0.10	0.610	−0.02	0.895	−0.06	0.750	−0.22	0.235
**pO_2_**	−0.12	0.522	0.39	0.029	0.48	0.006	−0.06	0.734	−0.09	0.645	0.06	0.750	−0.01	0.962	−0.22	0.237	−0.06	0.764	−0.16	0.385	−0.16	0.383
**pCO_2_**	0.00	0.992	−0.18	0.322	−0.09	0.615	0.01	0.944	0.02	0.935	0.06	0.746	0.30	0.101	0.18	0.321	0.13	0.476	0.33	0.066	0.39	0.028
**HCO_3_**	−0.03	0.855	−0.18	0.346	−0.12	0.534	0.11	0.570	0.10	0.599	0.05	0.778	0.19	0.303	0.24	0.192	0.15	0.415	0.34	0.058	0.33	0.068
**SpO_2_**	−0.18	0.336	0.40	0.028	0.37	0.039	−0.18	0.335	−0.14	0.464	−0.06	0.737	−0.07	0.710	−0.34	0.064	−0.24	0.186	−0.25	0.180	−0.27	0.148
**FEV1 Pre**	0.00	0.987	0.25	0.184	0.15	0.428	−0.40	0.026	−0.31	0.090	−0.21	0.266	−0.27	0.146	−0.40	0.024	−0.33	0.067	−0.21	0.254	−0.43	0.017
**FEV1 Post**	−0.04	0.845	0.23	0.204	0.11	0.573	−0.42	0.018	−0.37	0.042	−0.28	0.127	−0.37	0.042	−0.43	0.016	−0.40	0.026	−0.32	0.078	−0.56	0.001
**FVC Pre**	0.08	0.666	0.34	0.063	0.23	0.209	−0.32	0.082	−0.15	0.433	−0.12	0.504	−0.16	0.385	−0.34	0.065	−0.21	0.247	−0.11	0.561	−0.25	0.175
**FVC Post**	0.17	0.368	0.32	0.078	0.18	0.337	−0.28	0.123	−0.17	0.371	−0.14	0.461	−0.15	0.436	−0.35	0.052	−0.24	0.194	−0.10	0.586	−0.24	0.187
**V/C Pre**	−0.07	0.701	0.16	0.382	0.06	0.734	−0.34	0.065	−0.32	0.075	−0.21	0.256	−0.22	0.239	−0.33	0.069	−0.28	0.123	−0.18	0.322	−0.35	0.050
**V/C Post**	−0.18	0.331	0.17	0.364	0.12	0.525	−0.31	0.087	−0.25	0.167	−0.20	0.272	−0.22	0.233	−0.28	0.133	−0.22	0.237	−0.24	0.194	−0.40	0.024

r<0.3: Negligible correlation; r=0.3–0.5: Mild correlation; r=0.50.7: Moderate correlation; r>0.7: Strong correlation;

* Spearman rank correlation; - sign indicates an inverse correlation.

For spirometry parameters (Table 3), a mild linear correlation of Pre-FEV1 was observed with lower lobe and total average MLD while a mild linear correlation of post -FEV1 was observed with both coronal (p=0.042) and sagittal (p=0.001) lower lobes MLD.

**Table 3:** Correlation of Average MLD with Patient Characteristics and Pulmonary Function Parameters (Pearson bivariate)

	Average MLD					
	Upper Average MLD		Lower Average MLD		Total Average MLD	
	r	p	r	p	r	p
**Age**	−0.03	0.873	−0.12	0.533	−0.08	0.684
**Duration of disease**	0.06	0.741	−0.02	0.902	0.02	0.922
**Smoking index**	0.09	0.620	0.08	0.671	0.09	0.639
**Pack years**	0.13	0.479	0.11	0.561	0.12	0.513
MMRC*	0.25	0.182	0.12	0.524	0.18	0.322
GOLD Stage*	−0.07	0.692	−0.15	0.421	−0.12	0.536
**Hb**	0.14	0.469	0.17	0.358	0.16	0.400
**TLC**	0.12	0.523	0.02	0.920	0.07	0.714
**RBS**	−0.39	0.028	−0.34	0.059	−0.38	0.038
**Urea**	−0.15	0.405	−0.08	0.654	−0.12	0.518
**Creatinine**	−0.29	0.116	−0.24	0.190	−0.27	0.143
**Ph**	0.02	0.904	−0.13	0.482	−0.06	0.756
**pO_2_**	−0.10	0.609	−0.09	0.637	−0.09	0.616
**pCO_2_**	0.16	0.403	0.23	0.209	0.20	0.283
**HCO_3_**	0.20	0.291	0.21	0.248	0.21	0.258
**SpO_2_**	−0.22	0.240	−0.19	0.294	−0.21	0.257
**FEV1 Pre**	−0.33	0.069	−0.37	0.040	−0.36	0.047
**FEV1 Post**	−0.40	0.028	−0.47	0.007	−0.44	0.012
**FVC Pre**	−0.24	0.195	−0.21	0.252	−0.23	0.214
**FVC Post**	−0.24	0.202	−0.22	0.239	−0.23	0.211
**V/C Pre**	−0.29	0.114	−0.33	0.070	−0.32	0.083
**V/C Post**	−0.28	0.124	−0.31	0.093	−0.30	0.100

r<0.3: Negligible correlation; r=0.3–0.5: Mild correlation; r=0.50.7: Moderate correlation; r>0.7: Strong correlation;

* Spearman rank correlation

Also, there was a linear correlation between both pre (p=0.050) and post (p=0.024) FEV1/FVC with sagittal lower lobe MLD. In an attempt to quantify obstruction severity (FEV1) on the basis of MLD values (Table 4), a predictive model was prepared where FEV1% was considered as a dependent variable while MLD of coronal RUL, RLL, LUL, LLL and sagittal RUL, RLL, LUL, LLL were taken as an independent variable, MLD of sagittal RUL, sagittal RLL and sagittal LLL were found to be significantly associated with the dependent variable (p<0.05). The model had a fair explanatory ability (r2=0.439).

**Table 4:** Quantification of obstruction of severity (FEV1) on the basis of MLD values

Model		Unstandardized Coefficients		Standardized Coefficients	t	Sig.
		B	Std. Error	Beta		
1	(Constant)	−44.700	53.334	−	−0.838	0.410
	coronal RUL	0.184	0.127	0.544	1.455	0.158
	coronal RLL	−0.230	0.122	−0.793	−1.888	0.070
	coronal LUL	−0.106	0.115	−0.331	−0.919	0.367
	coronal LLL	0.219	0.128	0.694	1.719	0.098
	sag RUL	−0.350	0.138	−0.894	−2.537	0.018
	sag RLL	0.439	0.145	1.234	3.029	0.005
	sag LUL	0.173	0.147	0.493	1.179	0.249
	sag LLL	−0.218	0.104	−0.703	−2.103	0.045

a Dependent Variable: FEV1 %; r^2^=0.439; FEV_1_% = −44.700 + 0.184 CRUL − 0.230 CRLL − 0.106 CLUL + 0.219 CLLL − 0.350 SRUL + 0.439 RLL + 0.173 LUL − 0.218 LLL

## Discussion

At first glance, this study seems like one of many studies that assessed the characteristic HRCT features in patients with COPD. Only when we consider the clinical implications of this study do the differences become obvious. According to GOLD staging, the patients in our study were divided in 4 groups. Out of 35 patients enrolled in the study 17 patients were in GOLD stage II (n=17, 48.57%). COPD being a disease of old age and its association with prolonged duration of exposure to smoke and noxious particles, is a well known fact. Our findings were also in conformity with this data^10^. The mean age of patients in our study was 58.43±9.72 (ranging from 38 to 82 years).

The mean age was observed to be increasing with increasing GOLD stage. The duration of illness ranged from 1 to 20 years. Overall, mean duration of illness was 5.92±4.62 years and was found to be increasing with increasing GOLD stage. The mean duration of illness of stage IV patients was found to be lesser than stage III, which can be explained by the fact that patients with severe disease have frequent exacerbations, leading to steep fall in FEV1 and thus having shorter illness duration. Smoking as the prime and modifiable cause of COPD is an established fact^11^. In accordance, we also found a significant number of our patients to be smokers either current or previous. Almost all patients were smokers (n=31, 88.57%), while 4(11.43%) patients were nonsmokers. Among smokers, 13 (37.14%) patients had quit smoking. Statistically, significantly higher proportion of patients with smoking habit (smoker/ex-smokers) had Stage IV of COPD as compared to stage I, II and III of COPD (p=0.053). Mean pack years was 22.76 pack years (range=6.5–60 pack years).

Among clinical features, breathlessness was found to be the most common presenting complaint and was invariably present in all the patients. 26(74%) patients were coughing and 23(65.71%) patients had expectoration. These findings were similar to the data from the Third National Health and Nutrition Examination Survey (NHANES III) which showed that a large majority of patients with severe COPD (FEV1<50% of predicted) may be asymptomatic. The symptoms reported most frequently were wheezing and shortness of breath^12^. While comparing hematological and biochemical parameters, 4 (11.4%) patients had hemoglobin percentage >17 gm/dl, thus classifying as polycythemia^13^, most probable cause being chronic hypoxia. According to the WHO definition, anemia in males is classified as Hb<13 g/dl^14^. In our study, 15 (42.88%) patients qualified as anemia according to this definition. This finding can be explained by the fact that the inflammatory mediators in circulation in patients with COPD lead to either initiation or worsening of co-morbidities like normocytic anemia, metabolic syndrome and diabetes. The values of serum creatinine was found to be significantly higher in stage IV as compared to other stages (p=0.016), the most probable causes being increasing age, recurrent infections and the use of antibiotics. On evaluating oxygenation status among COPD patients, 19 (54.28%) patients had hypoxia, while 12 (34.28%) patients had hypercapnia (pCO_2_ >45 mm Hg).For all the other parameters, the difference among different stages was not significant statistically (p>0.05).

Although a chest radiograph is not essential in the diagnosis of COPD, yet it has an important role. There are certain radiological features suggestive of emphysema, namely, flattening of diaphragm, pruning of vascular markings, tubular heart, increased retrosternal air space, among others and certain features suggestive of chronic bronchitis, such as prominence of markings (dirty chest), cardiomegaly and prominent pulmonary trunk. But, chest X ray has limited sensitivity and specificity^15^,^16^. In our study, 4 (11.42%) patients had bullae detected on chest X ray, while HRCT helped detect bullae in 10 (28.57%) patients. On evaluating the level of agreement between chest X ray and HRCT in diagnosing bullae, it was found to be of moderate order (k>0.2) which was also significant statistically (k=0.317; p=0.029). Similarly, bronchiectasis was reported in only 2 ( 5.71%) patients on CXR, while 19 (54.28%) patients had bronchiectasis on HRCT scan. Decrease in size as well as number of pulmonary vessels presents as vascular attenuation. It is a common accompaniment to emphysema and airway destruction, and is an important HRCT feature. Various studies have demonstrated significant correlation between vascular attenuation and FEV1% and dyspnea scale^17^,^18^. Vascular attenuation was the most common HRCT finding (n=31, 88.57%), although no correlation with disease severity was found.

Emphysema on HRCT is defined as areas of abnormally low attenuation. In our study, emphysema was noted in 31/35 patients(88.57%), which is comparable to the findings of the study by Gupta et al,^18^ who reported that 25 patients had at least one type of emphysema (sensitivity 62.5 %). Bullae are a common associated feature of emphysema, which develop as a result of progressive destruction of respiratory bronchioles and alveoli. This has an important impact on both the symptomatology and the overall morbidity and prognosis. Sometimes the mode of therapy may totally change towards a surgical option. Bullectomy may be beneficial in patients with large bullae and predominantly bullous emphysema.^19^ This can be detected with confidence only by HRCT, it assesses the extent of bullous disease and the degree of compression and emphysema in the remaining lung parenchyma^20^. Bullae were detected in 10 (28.57%) patients in our study population. Similar findings have been found by Mostafa et al also who reported bullae in 7/50(14%) patients^21^. Other features detected were cyst in 6 (11.14%) patients, which is similar to that reported by Aydin et al^22^, found cyst formation in 4 (8%) subjects. Our study revealed bronchiectasis in 19 (54.28%) patients. Also, patients with bronchiectasis were more often smokers and had lower FEV1. This high percentage of incidental diagnosis of bronchiectasis in patients of COPD raises a number of questions, namely, whether this bronchiectasis phenotype can be included in the spectrum of chronic bronchitis and emphysema or whether COPD is late sequelae of primary bronchiectasis. This warrants further large scale studies.

Patients with COPD tend to have shorter transverse than AP diameter of the trachea, leading to lower tracheal index. Tracheal index has been significantly correlated with the FRC values^23^. Tracheal index had significant inverse correlations with duration of illness, smoking pack years, and dyspnea scale; and had direct correlations with FEV1, PEFR, FEV1/FVC ratio, and FEV1/SVC ratio^18^. Our study found a mean tracheal index of 0.96 (range=0.36–1.95). Saber sheath trachea (TI<0.67) was seen in 2 (5.71%) patients. Tracheal index was found to be decreasing with increasing disease severity (GOLD stage) and this was statistically significant (p=0.037). The normal thoracic cage ratio is 0.71, with the transverse thoracic diameter being more than the AP diameter. But, in COPD patients, due to lung hyperinflation, the AP diameter increases, causing the thoracic cage ratio to increase. In our study, mean TCR at carina was 0.77, while mean TCR at a level 5 cm below the carina was 0.69. TCR at carina >0.75 (suggestive of hyperinflation) was seen in 11(31.4%) patients, while barrel shaped chest (TCR>0.9) was seen in 6(17.1%) patients. TCR at 5 cm below carina >0.75 was seen in 12 (34.28%) patients while TCR>0.9 was seen in 5 (14.28%) patients. We also found significant inverse correlation between TCR at 5 cm below carina and GOLD stage (p=0.042). Our findings are exactly opposite to that reported by Gupta et al^18^ who found that both these values had direct correlations with duration of illness, smoking pack years and dyspnea scale; and had inverse correlations with FEV1, FEV1/FVC ratio and FEV1/SVC ratio. This discrepancy can be explained by the fact that the GOLD staging uses only spirometric indices as disease severity. If we consider the combined assessment score which includes the symptoms (MMRC) as well as exacerbation history, we find MMRC increasing with severity. Thus we need a broader parameter for disease severity assessment. Another sign of hyperinflation in patients with COPD is the increased distance from the posterior sternal surface to the anterior margin of the ascending aorta^24^, depicted by the SAD. Mean SAD was 2.87 cm (range=1.13–4.26) in our study. SAD of >4 cm was seen in 2 (5.71%) patients. In our study, no correlation was found between SAD and severity of disease, the cause being that most of the patients reporting to our tertiary care centre present with exacerbation and it can be assumed that these patients having frequent exacerbations have rapid decline in FEV1 and proceed from stage I to IV, without developing the physical signs of hyperinflation.

Various studies have found correlation between mean lung density and FEV1%^25^,^26^. The mean lung density of a normal lung without any ventilation defect has been shown to be −800 HU. As the severity of emphysema goes on increasing, the MLD decreases. In our study, mean upper lobe MLD was −839.27 HU, mean lower lobe MLD was −834.91 HU and the mean MLD was −837.08 HU. For spirometry parameters, a mild linear correlation of pre-FEV1 was observed with lower lobe and total average MLD while a mild linear correlation of post-FEV1 was observed with both coronal (p=0.042) and sagittal (p=0.001)lower lobes MLD. Also, there was a linear correlation between both pre (p=0.050) and post (p=0.024) FEV1/FVC with sagittal lower lobe MLD. These findings are consistent with the findings of Torres et al^27^, who reported that the lower lobe MLD on inspiration and expiration were lower in patients with very severe or severe COPD than in those with moderate disease. Hermans et al also found that the mean MLD values in patients having COPD were much less than the lung density in normal persons^28^.

## Conclusion

The current evaluation of COPD patients requires only Spirometry. But, quantifying this complex and multisystem disease just on the basis of measuring airway obstruction is not justified and not feasible in all cases. HRCT may be an important additional tool in the holistic evaluation of this disease and it can be a substitute to Spirometry in certain situations. Our study found that HRCT can well be correlated with the spirometric and clinical features and the level of obstruction can be indirectly derived from it by measuring the MLD. Further large scale studies are warranted to consolidate these findings.

## Footnotes

[a1] In global scenario, Spirometry is expensive diagnostic tool for COPD diagnosis, but in Indian scenario it easily available at low cost, so I wrote Spirometry is inexpensive, easily reproducible and readily available test Spirometry

[a2] Thewritten informed consent was obtained fulfilling the inclusion and exclusion criteria of the study. The study was approved by the institutional ethics committee.

[a3] Spirometry performed according to GOLD criteria (COPD was defined by a post-bronchodilator FEV1/FVC ratio < 0.70. COPD severity was determined by the GOLD criteria. Subjects with a post-bronchodilator FEV1/FVC ratio ≥ 0.70 PubMed were considered not to have COPD) in the study.

[a4] Exclusion criteria

[SK5] Included in statistical section
